# Significant Prognostic Value of the Autophagy-Related Gene P4HB in Bladder Urothelial Carcinoma

**DOI:** 10.3389/fonc.2020.01613

**Published:** 2020-08-13

**Authors:** Lei Lyu, Wei Xiang, Fuxin Zheng, Tao Huang, Yan Feng, Jingdong Yuan, Chuanhua Zhang

**Affiliations:** ^1^Department of Urology, Wuhan No.1 Hospital, Huazhong University of Science and Technology, Wuhan, China; ^2^Department of Pathology, Wuhan No.1 Hospital, Huazhong University of Science and Technology, Wuhan, China

**Keywords:** bladder urothelial carcinoma, autophagy-related genes, prognosis, biomarker, prolyl 4-hydroxylase, beta polypeptide

## Abstract

While hundreds of consistently altered autophagy-related genes (ARGs) have been identified in cancers, their prognostic value in bladder urothelial carcinoma (BUC) remains unclear. In the present study, we collected 232 ARGs from the Human Autophagy Database (HADb), and identified 37 differentially expressed ARGs in BUC based on The Cancer Genome Atlas (TCGA) database. Kaplan-Meier survival analysis based on the Gene Expression Profiling Interactive Analysis (GEPIA) database revealed that among the 37 differentially expressed ARGs, prolyl 4-hydroxylase, beta polypeptide (P4HB), and regulator of G protein signaling 19 (RGS19) were significantly negatively correlated with overall survival (OS) and disease-free survival (DFS). Overexpression of P4HB and RGS19 in BUC was further validated using independent data sets, including those from the Oncomine and Gene Expression Omnibus (GEO) databases. cBioPortal and UALCAN analyses indicated that altered P4HB and RGS19 mRNA expression was significantly associated with mutations and clinical characteristics (nodal metastasis and cancer stage). Moreover, co-expression network analysis and gene set enrichment analysis (GSEA) predicted that the potential functions of P4HB and RGS19 are involved in the endoplasmic reticulum (ER) stress response, cytokine-mediated signaling pathway and inflammatory response. More importantly, multivariate Cox proportional hazards regression analysis demonstrated that P4HB, but not RGS19, is an independent and unfavorable BUC biomarker based on clinical characteristics (age, gender, cancer stage, and pathological TNM stage). Finally, we validated that the mRNA and protein expression levels of P4HB were upregulated in four bladder cancer cell lines (T24, J82, EJ, and SW780) and found that knockdown of P4HB dramatically inhibited the invasion and proliferation of bladder cancer cells. In summary, our study screened ARGs and identified P4HB as a biomarker that can predict the progression and prognosis of BUC and may provide a better understanding of the autophagy regulatory mechanisms involved in BUC.

## Introduction

Bladder cancer is the most common malignancy of the urinary system and the leading cause of cancer-associated mortality in the elderly population of China (1). The pathological types of bladder cancer mainly include bladder urothelial carcinoma (BUC), bladder squamous cell carcinoma and bladder adenocarcinoma. The most common pathological type of bladder cancer is BUC, which has unique characteristics, such as drug resistance, a high recurrence rate, a higher frequency of metastasis, and poor prognosis (2, 3). However, traditional clinicopathological risk factors could not sufficiently identify BUC patients with high risk and predict the prognosis of BUC. Recently, molecular biomarkers have been shown to aid the diagnosis and therapy and guide the prediction of the prognosis for BUC (4). For example, Chang et al. reported that BCAT1 is a potential diagnostic and prognostic marker for BUC patients (5). Zhang et al. revealed that high expression of HEF1 is associated with advanced stage and shortened progression-free survival poor for BUC patients (6). However, the clinical significance of these potential biomarkers and functionally important genes were not definitively verified because of a lack of larger clinical cohorts. Therefore, it is necessary to identify valuable biomarkers using large clinical samples and further investigate the molecular mechanisms involved in the development of BUC.

Currently, autophagy has been extensively studied and proposed as a significant factor in tumor development (7–9). Autophagy has both protective and harmful biological functions, including pro-, or antitumor effects, depending on the tumor microenvironment. On the one hand, autophagy enhanced cancer cells to tolerate stress responses, including a hypoxic microenvironment, starvation, and chemotherapy (10, 11). On the other hand, autophagy plays a critical role in damage mitigation in response to stress that can inhibit tumor progression by degrading defective proteins and organelles and by preventing abnormal protein accumulation (12). Regarding BUC, autophagy-targeted therapy has recently been considered a valuable strategy. It was recently reported that autophagy-related gene (ARG) levels are associated with the chemosensitivity of BUC and markedly affect the regulation of invasion (13). However, the role and mechanisms of autophagy remain far from clear. In the present study, we used bioinformatics analysis to investigate the expression of variations in 223 ARGs in BUC and to explore their potency as biomarkers. We finally identified prolyl 4-hydroxylase, beta polypeptide (P4HB) as a novel potential biomarker for BUC diagnosis and prognosis. Moreover, we demonstrated that knockdown of P4HB in human bladder cancer cells *in vitro* dramatically inhibited cancer cell invasion. The present study developed an ARG into a potential biomarker that provides a deeper understanding of the mechanism of autophagy in BUC.

## Materials and Methods

### Collection of ARGs

We first collected 232 ARGs from the Human Autophagy Database (HADb^1^). Subsequently, we downloaded the RNA expression profiles (RNA-Seq2 level 3 data; platform: Illumina HiSeq 2000, through Mar 2020) and clinical data of BUC patients from The Cancer Genome Atlas (TCGA) database^2^. TCGA provided the mRNA expression data of 430 samples (411 BUC samples and 19 normal bladder tissue samples).

### Functional Enrichment Analysis

The GO database^3^ was used to analyze differentially expressed ARGs. The molecular functions (MFs), cellular components (CCs), and biological processes (BPs) of differentially expressed ARGs were identified. The potential functions of the differentially expressed ARGs involved in signaling pathways were analyzed using the Kyoto Encyclopedia of Genes and Genomes (KEGG)^4^ and Reactome^5^. The relationships among the enriched clusters from the GO and signaling pathway analyses were visualized using Metascape^6^ (14). In addition, gene set enrichment analysis (GSEA) was performed to evaluate the correlation between P4HB or regulator of G protein signaling 19 (RGS19) expression and BUC-associated pathways. The detailed protocol for GSEA is available on the Broad Institute Gene Set Enrichment Analysis website^7^ (15).

### Survival Analysis

Kaplan-Meier survival curves were generated to evaluate the prognostic value of the ARGs using the online database Gene Expression Profiling Interactive Analysis (GEPIA2)^8^ (16). For the overall survival (OS) and disease-free survival (DFS) analyses, the BUC patients were divided into 2 groups according to the median expression of the mRNAs (high vs. low). The survival curves of samples with low mRNA expression and high mRNA expression were compared using the log-rank test. *P* < 0.05 indicated statistically significant differences. In addition, multivariate Cox proportional hazards regression analysis was performed to determine the P4HB, RGS19, and clinical features that were significantly associated with OS.

### Oncomine Database and GEO Database Analyses

The mRNA expression of P4HB and RGS19 in BUC was analyzed within the Oncomine database^9^. The thresholds were restricted as follows: *P*-value: 0.0001, fold change: 1.5, gene ranking: all, analysis type: cancer vs. normal, and data type: mRNA. This analysis drew on a series of BUC studies, including the Modlich, Sanchez-Carbayo, Dyrskjot and Lee studies (17–20). In addition, the mRNA expression of P4HB and RGS19 was validated in three independent Gene Expression Omnibus (GEO)^10^ data sets (GSE13507, GSE52519, and GSE37815) using GEO2R. The gene expression profiling databases were obtained from GEO.

### UALCAN and cBioPortal Analyses

UALCAN is an interactive web portal for facilitating tumor subgroup gene expression and survival analyses^11^ (21). We used UALCAN analysis to estimate the P4HB and RGS19 expression levels based on the clinical features (gender, age, cancer stage, and nodal metastasis status) of BUC from TCGA data sets. The cBioPortal for cancer genomics^12^ is an exploratory analysis tool for exploring multidimensional cancer genomics data sets. The frequency of P4HB and RGS19 alterations (amplification, deep deletion, and missense mutation) and copy number variations (CNVs) were obtained from Genomic Identification of Significant Targets in Cancer (CISTC). In addition, the OncoPrint sub-tool of cBioPortal was also utilized to analyze the integrated status of CNVs for P4HB and RGS19.

### LinkedOmics Analysis

LinkedOmics^13^ is a publicly available portal that includes multiomics data from all 32 TCGA cancer types (22). The LinkFinder module of LinkedOmics was used to analyze the differentially expressed genes correlated with P4HB or RGS19 in BUC from TCGA cohort. We constructed a co-expression network based on the Pearson correlation coefficient (| cor| > 0.35, *P* < 0.05) between P4HB or RGS19 and the mRNAs to predict the potential targets of P4HB or RGS19. In addition, we used GeneMANIA^14^ (23) to visualize the gene network of P4HB and RGS19.

### Cell Culture

Three human BUC lines (T24, J82, and SW780) and a human normal uroepithelial cell line (SV-HUC-1) were obtained from the American Type Culture Collection (ATCC, Manassas, VA, United States). The BUC EJ cell line was obtained from the Institute of Biochemistry and Cell Biology of Chinese Academy of Sciences (Shanghai, China). Cells were cultured in RPMI-1640 medium supplemented with 10% fetal bovine serum (Thermo Scientific HyClone, Logan, UT, United States), 100 U/ml penicillin, and 100 μg/ml streptomycin at 37°C and 5% CO_2_.

### RNA Interference and Transfection

The mRNA sequence of the P4HB gene was obtained from GenBank (NM_000918), and the targeting sequence was designed using an RNAi algorithm available online^15^. The P4HB-specific siRNA (5′-GTCCTCTTTAAGAAGTTTGATGA-3′) and a nonsense siRNA [negative control siRNA (NC siRNA)] were synthesized and purified by GenePharma (Shanghai, China). T24 and EJ cells were transfected with siRNAs using Translipid reagent (TransGen, Beijing, China) according to the manufacturer’s protocol.

### qRT-PCR

Total RNA was extracted using the TRIzol Reagent kit (Invitrogen, Carlsbad, CA, United States) and was reverse transcribed into cDNA by using PrimeScript RT-polymerase (TaKaRa, Dalian, China). Real-time PCR was performed on the cDNA templates using specific primers (Sangon, Shanghai, China) and SYBR master mix (TaKaRa, Dalian, China). The relative mRNA expression levels of P4HB were calculated as a ratio normalized to GAPDH expression. Comparative quantification was performed using the 2^–Δ^ ^Δ^ ^*Ct*^ method. The sequences of the specific primers used in the present study were as follows: P4HB (NM_000918), forward primer 5′-TCACATCCTGCTGTTCTTG-3′, reverse primer 5′-ACTTGGTCATCTCCTCCTC-3′; and GAPDH (NM_002046), forward primer 5′-TGAAGGTCGGAGTCAACGG-3′, and reverse primer 5′-CCTGGAAGATGGTGATGGG-3′.

### Western Blot

Bladder urothelial carcinoma tissues and cells were lysed with RIPA buffer containing protease inhibitor (Thermo Fisher Scientific, Waltham, MA, United States). Proteins were quantified and resolved by 12% SDS-PAGE and electrotransferred to polyvinylidene difluoride (PVDF) membranes (Millipore Bedford, MA, United States). Then, the cells were incubated with 5% skim milk at room temperature for 30 min and with primary antibodies against P4HB (Cell Signaling Technology, Beverly, MA, United States) overnight at 4°C. Then, the cells were incubated with horseradish peroxidase (HRP)-conjugated secondary antibodies (Santa Cruz, CA, United States) and detected using a chemiluminescence method (ECL, Thermo Fisher Scientific, Waltham, MA, United States) according to the manufacturer’s instructions. An anti-GAPDH (Santa Cruz, United States) antibody was used as a control.

### Cell Viability Assays

The effect of P4HB-specific siRNA on cell viability was tested using an MTT assay (Beyotime Institute Biotechnology, Shanghai, China) according to the manufacturer’s instructions. Briefly, T24 and EJ cells at 5 × 10^3^ cells per well were cultured in 96-well plates and transfected with 100 nM NC siRNA and P4HB-specific siRNA for the indicated periods. Then, 20 μl MTT (5 mg/ml) was added to each well, and the cells were incubated for an additional 4 h. The relative levels of cell proliferation in each group of cells compared to that in control cells were calculated, and the control cells were designated to have a cell proliferation rate of 100%. All experiments were repeated at least three times.

### Invasion Assays

Cell invasion assays were performed in a Boyden chemotaxis chamber (Costar, United States). Briefly, 5 × 10^4^ cells resuspended in serum-free RPMI 1640 medium were placed in the upper chamber, while the lower chamber was filled with 10% FBS-containing RPMI 1640. After incubation for 24 h, the cells in the upper chamber were removed, and the cells at the bottom of the polycarbonate membrane were fixed and stained with 0.1% crystal violet. The number of invading cells was counted in three randomly chosen fields under the microscope.

## Results

### Differentially Expressed ARGs in TCGA

**FIGURE 1:**
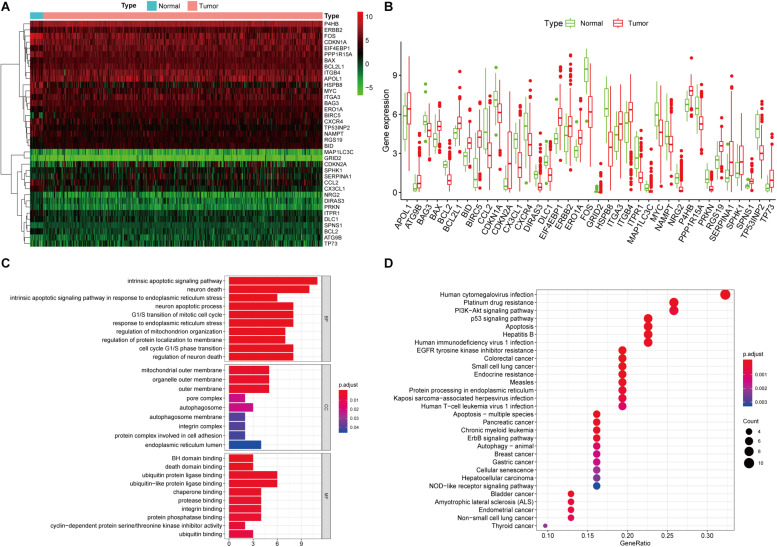
Differentially expressed ARGs and functional annotation. **(A)** Heatmap of the differentially expressed ARGs. **(B)** The differentially expressed ARGs exhibited as a histogram. **(C)** GO enrichment analysis of differentially expressed ARGs. **(D)** KEGG pathway enrichment analysis of differentially expressed ARGs. The top 30 enriched pathways are shown.

A total of 232 ARGs were collected from the HADb. The expression level of each of the 232 ARGs was compared between BUC and normal bladder tissues in the TCGA dataset, which contained 411 BUC samples and 19 adjacent non-tumor bladder tissue samples. Thirty-seven differentially expressed ARGs were identified, among which 18 were upregulated and 19 were downregulated (Figures 1A,B). All of the differentially expressed genes are listed in Table 1.

**TABLE 1:** The differentially expressed ARGs in BUC (Tumor vs. Normal).

Gene symbol	Ensembl ID	logFC	Regulation	*P*-value	FDR
CDKN2A	ENSG00000147889	4.19	Up	2.49E-03	5.39E-03
SERPINA1	ENSG00000197249	2.85	Up	1.16E-03	3.12E-03
BIRC5	ENSG00000089685	2.09	Up	8.84E-09	1.59E-07
TP73	ENSG00000078900	1.96	Up	2.51E-04	8.50E-04
ATG9B	ENSG00000181652	1.70	Up	8.09E-04	2.26E-03
EIF4EBP1	ENSG00000187840	1.68	Up	1.27E-09	3.51E-08
SPHK1	ENSG00000176170	1.52	Up	1.64E-02	2.96E-02
ERBB2	ENSG00000141736	1.45	Up	3.68E-03	7.73E-03
BID	ENSG00000015475	1.35	Up	7.39E-10	2.85E-08
ITGA3	ENSG00000005884	1.22	Up	4.54E-03	9.13E-03
APOL1	ENSG00000100342	1.18	Up	2.55E-02	4.28E-02
RGS19	ENSG00000171700	1.16	Up	2.44E-07	2.24E-06
ERO1A	ENSG00000197930	1.10	Up	3.07E-07	2.58E-06
P4HB	ENSG00000185624	1.09	Up	1.19E-09	3.51E-08
SPNS1	ENSG00000169682	1.09	Up	1.68E-08	2.50E-07
ITGB4	ENSG00000132470	1.06	Up	7.56E-04	2.16E-03
BCL2L1	ENSG00000171552	1.03	Up	5.10E-07	4.10E-06
BAX	ENSG00000087088	1.00	Up	9.04E-09	1.59E-07
MAP1LC3C	ENSG00000197769	–1.01	Down	4.41E-05	1.89E-04
GRID2	ENSG00000152208	–1.01	Down	8.41E-08	9.55E-07
BAG3	ENSG00000151929	–1.12	Down	1.05E-04	4.14E-04
NAMPT	ENSG00000105835	–1.15	Down	7.33E-03	1.39E-02
PPP1R15A	ENSG00000087074	–1.23	Down	5.54E-06	3.45E-05
CX3CL1	ENSG00000006210	–1.25	Down	2.01E-07	1.94E-06
CXCR4	ENSG00000121966	–1.27	Down	2.19E-03	4.92E-03
DIRAS3	ENSG00000162595	–1.33	Down	2.18E-08	3.01E-07
CDKN1A	ENSG00000124762	–1.35	Down	2.51E-04	8.50E-04
BCL2	ENSG00000171791	–1.36	Down	5.96E-10	2.85E-08
DLC1	ENSG00000164741	–1.40	Down	1.66E-07	1.68E-06
MYC	ENSG00000136997	–1.44	Down	1.71E-05	9.72E-05
NRG2	ENSG00000158458	–1.73	Down	9.04E-09	1.59E-07
TP53INP2	ENSG00000078804	–1.97	Down	1.90E-10	2.05E-08
ITPR1	ENSG00000150995	–2.40	Down	1.12E-08	1.80E-07
PRKN	ENSG00000185345	–2.43	Down	3.46E-10	2.23E-08
HSPB8	ENSG00000152137	–2.83	Down	2.91E-09	7.02E-08
CCL2	ENSG00000108691	–2.89	Down	1.57E-06	1.01E-05
FOS	ENSG00000170345	–2.94	Down	2.13E-10	2.05E-08

### Enrichment Analyses of Differentially Expressed ARGs

We performed GO and KEGG pathway enrichment analyses to determine the potential functions of these dysregulated ARGs in the development of BUC. The GO plot analysis indicated that in the BPs, these genes were associated with the intrinsic apoptotic signaling pathway, as well as with the response to ER stress and the cell cycle. In terms of the CCs, these genes were involved in the autophagosome, mitochondrial outer membrane and cell adhesion. With regard to MF, these genes participated in certain key functions, such as ubiquitin protein ligase binding and protein phosphatase binding. The significant KEGG pathways in which the differentially expressed ARGs were enriched were mainly the p53 signaling pathway, apoptosis, autophagy and PI3K-Akt signaling pathway. In addition, the KEGG pathway enrichment analysis indicated that these genes were associated with multiple cancers, such as bladder cancer, pancreatic cancer, chronic myeloid leukemia, and breast cancer, which identified the major roles of these genes in tumorigenesis and development (Figures 1C,D).

### Kaplan-Meier Survival Analyses of Differentially Expressed ARGs

**FIGURE 2:**
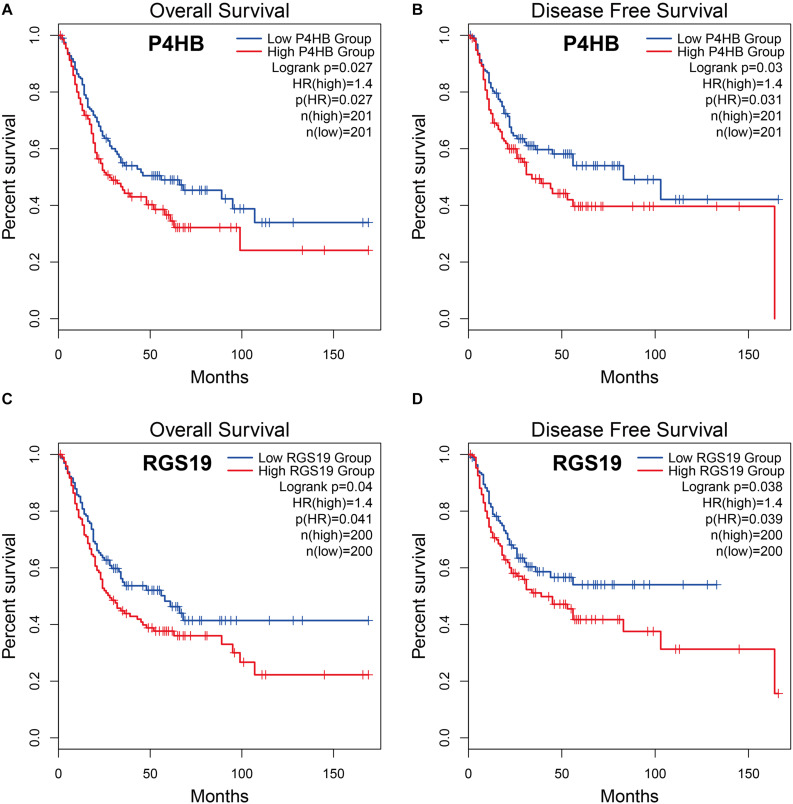
Relationship between P4HB and RGS19 expression and prognosis in BUC patients. **(A,B)** Kaplan-Meier survival curves of OS and DFS comparing patients with high (red) and low (blue) expression of P4HB in BUC. **(C,D)** Kaplan-Meier survival curves of OS and DFS comparing patients with high (red) and low (blue) expression of RGS19 in BUC.

To explore whether the differentially expressed ARGs were correlated with survival time, BUC cases were divided into two groups (low vs. high groups) according to the expression level of ARGs, and each group was analyzed by Kaplan-Meier survival analysis using the GEPIA database. The results showed that among the 37 differentially expressed ARGs, only two ARGs (P4HB and RGS19) were significantly negatively correlated with OS and DFS (log-rank test, *P* < 0.05), suggesting that the expression levels of P4HB (Figures 2A,B), and RGS19 (Figures 2C,D) were closely related to BUC prognosis. In the following studies, we focused on investigating the biological role of these two genes in BUC.

### Validation of the mRNA Expression of P4HB and RGS19 in the Oncomine and GEO Databases

**FIGURE 3:**
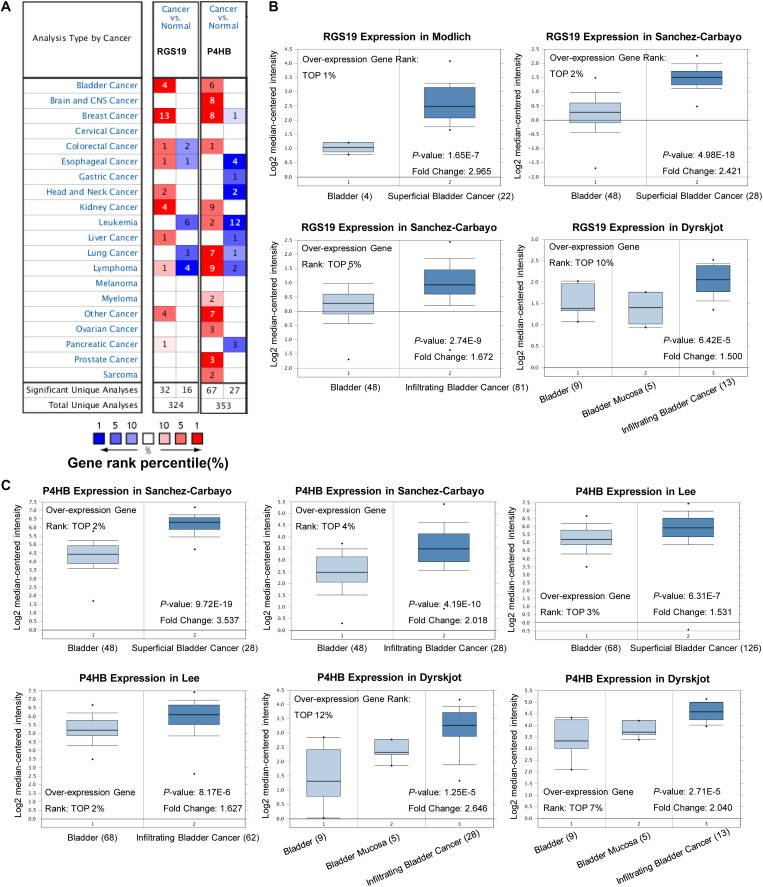
Expression patterns of P4HB and RGS19 in the Oncomine database. **(A)** The transcription levels of P4HB and RGS19 in different types of cancers. **(B)** Box plot showing the RGS19 mRNA levels in the Modlich, Sanchez-Carbayo, and Dyrskjot bladder cancer data sets. **(C)** Box plot showing the P4HB mRNA levels in the Sanchez-Carbayo, Lee and Dyrskjot bladder cancer data sets.

To validate the difference in P4HB and RGS19 expression in tumor and normal tissues, especially in BUC, the P4HB and RGS19 mRNA levels in different tumors and normal tissues of multiple cancer types were analyzed using the Oncomine database. The analysis results revealed that P4HB expression was higher in bladder, brain, breast, kidney, lung, prostate, ovarian cancers, and lymphoma tumors than in normal tissues. RGS19 expression was higher in bladder, breast, and kidney cancer tissues than in normal tissues. However, expression of P4HB in esophageal, head, and neck cancers and leukemia was lower than expression in other cancers in some data sets. Moreover, expression of RGS19 was lower in leukemia, lung cancer, and lymphoma than in other cancers (Figure 3A). These results further confirmed that both the P4HB and RGS19 expression levels were higher in BUC than in normal bladder tissue (Figures 3B,C). The expression of P4HB and RGS19 was further tested in three independent GEO data sets (GSE13507, GSE52519, and GSE37815) using GEO2R. Consistent with the results of TCGA database and Oncomine database, the mRNA expression levels of P4HB and RGS19 in BUC were upregulated compared with the expression levels in normal bladder tissue (Table 2).

**TABLE 2:** The mRNA expression of P4HB and RGS19 in the GEO databases.

Gene symbol	Ensembl ID	GEO	Platform	Platform ID	Fold change	*P* value
RGS19	ENSG00000171700	GSE13507	GPL6102	ILMN_1677085	1.26	3.56E-02
		GSE52519	GPL6884		2.15	4.36E-03
		GSE37815	GPL6102		1.24	1.30E-01
P4HB	ENSG00000185624	GSE13507	GPL6102	ILMN_1719303	1.89	3.31E-04
		GSE52519	GPL6884		1.75	2.36E-02
		GSE37815	GPL6102		1.98	8.07E-05

### Genomic Alterations of P4HB and RGS19 in BUC

**FIGURE 4:**
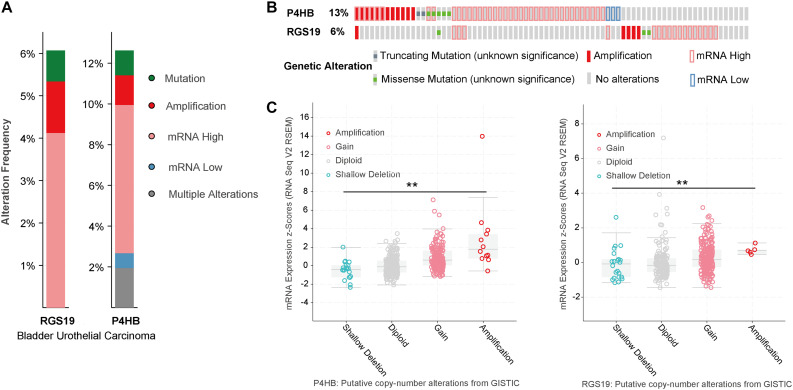
Alteration frequency and mRNA expression of P4HB and RGS19 in BUC (cBioPortal). **(A)** Summary of alterations in P4HB and RGS19. **(B)** Oncoprint represents the distribution and proportion of samples with different types of alterations in P4HB and RGS19. **(C)** The relationship of P4HB or RGS19 mRNA expression and gene mutations. **P < 0.001.

We analyzed the genomic alterations of P4HB and RGS19 by using the cBioPortal online database for BUC. P4HB and RGS19 were altered in 52 (12.62%) and 25 (6%) of the 413 BUC patients, respectively. For P4HB, these alterations were mRNA upregulation in 30 cases (7.28%), mRNA downregulation in 3 cases (0.73%), amplification in 6 cases (1.46%), mutation in 5 cases (1.21%), and multiple alterations in 8 cases (1.94%). For RGS19, these alterations were mRNA upregulation in 17 cases (4.13%), amplification in 5 cases (1.21%), and mutation in 3 cases (0.73%; Figures 4A,B). Next, we analyzed the mutant mRNA expression of P4HB and RGS19. There was a significant difference in the P4HB and RGS19 expression levels between shallow deletion and amplification in the copy number alteration status in BUC, according to TCGA database analysis (Figure 4C).

### Relationship Between the mRNA Levels of P4HB or RGS19 and the Clinicopathological Features of Patients With BUC

**FIGURE 5:**
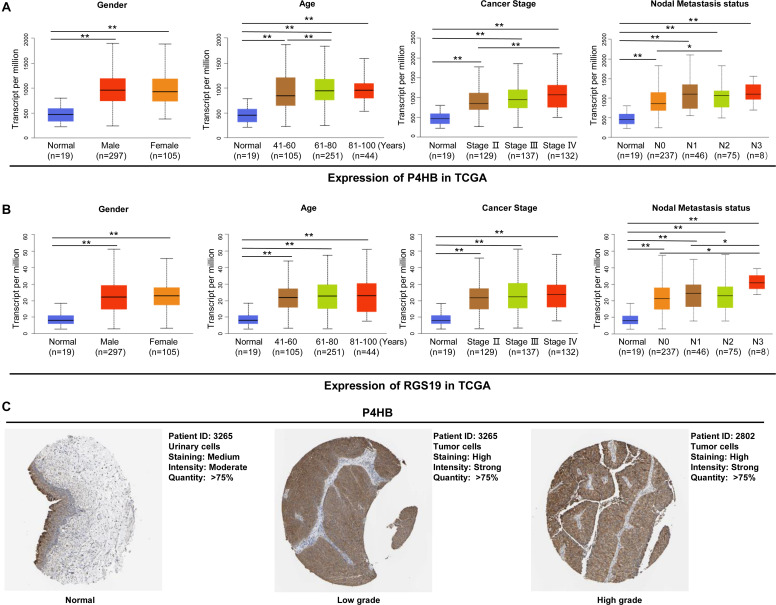
P4HB and RGS19 mRNA and protein expression in subgroups of patients with BUC. Boxplot showing the relative expression of P4HB **(A)** and RGS19 **(B)** mRNA in normal bladder tissues and subgroups of BUC samples stratified based on gender, age, cancer stage, and nodal metastasis status. **(C)** The representative protein expression of P4HB in normal bladder tissues, low grade and high grade BUC. **P < 0.01, *P < 0.05.

To elucidate the relevance of P4HB and RGS19 with respect to tumor progression, we further performed a subgroup analysis of multiple BUC clinicopathological features. The analysis included stage II–IV BUC cases (stage I was omitted due to only two cases being available from TCGA database). The transcription levels of P4HB and RGS19 were significantly higher in the tumor tissues than in the non-cancerous bladder tissues in subgroup analyses based on gender, age, cancer stage and nodal metastasis status (Figures 5A,B). In addition, we analyzed the protein expression levels of P4HB and RGS19 in BUC using The Human Protein Atlas (THPA) database^16^. The results revealed that the protein expression levels of P4HB in BUC tissue were also upregulated compared with those in normal bladder tissue. However, the RGS19 protein was not detected in the BUC tissue or normal bladder tissue (Figure 5C).

### Biological Interaction Network of P4HB and RGS19 in BUC

**FIGURE 6:**
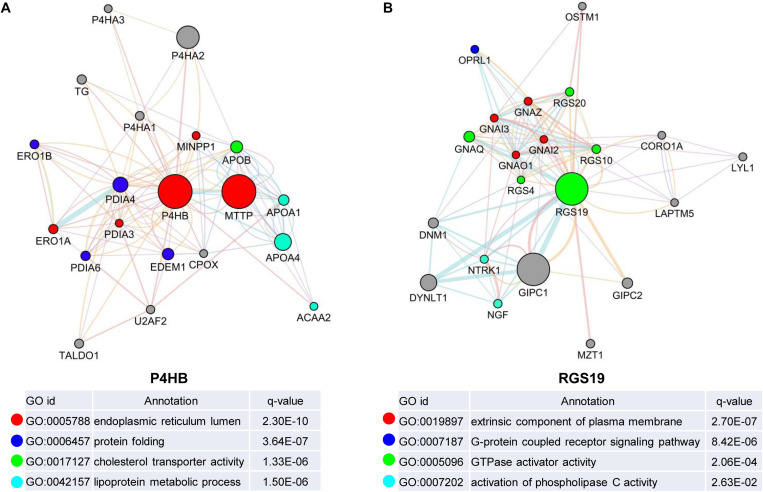
Identification of the biological functions of P4HB and RGS19 using the GeneMANIA database. Biological function analyses revealed the gene set that was enriched in the target network of P4HB **(A)** and RGS19 **(B)**. The different colors for the network nodes indicate the biological functions of the enriched set.

**FIGURE 7:**
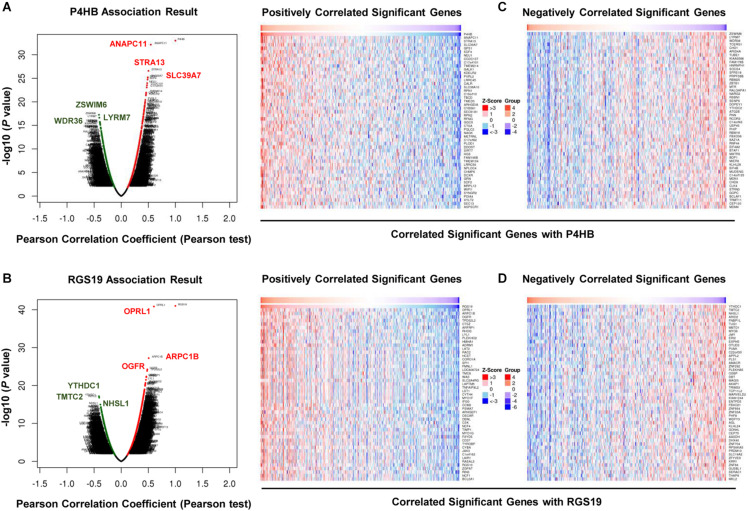
Differentially expressed gene correlations with P4HB or RGS19 in BUC (LinkedOmics). A Pearson test was used to analyze the correlation between P4HB **(A)** or RGS19 **(B)** and differentially expressed genes in BUC. Heat maps showing genes positively and negatively correlated with P4HB **(C)** or RGS19 **(D)** in BUC (top 50 genes). Red indicates positively correlated genes, and green indicates negatively correlated genes.

**FIGURE 8:**
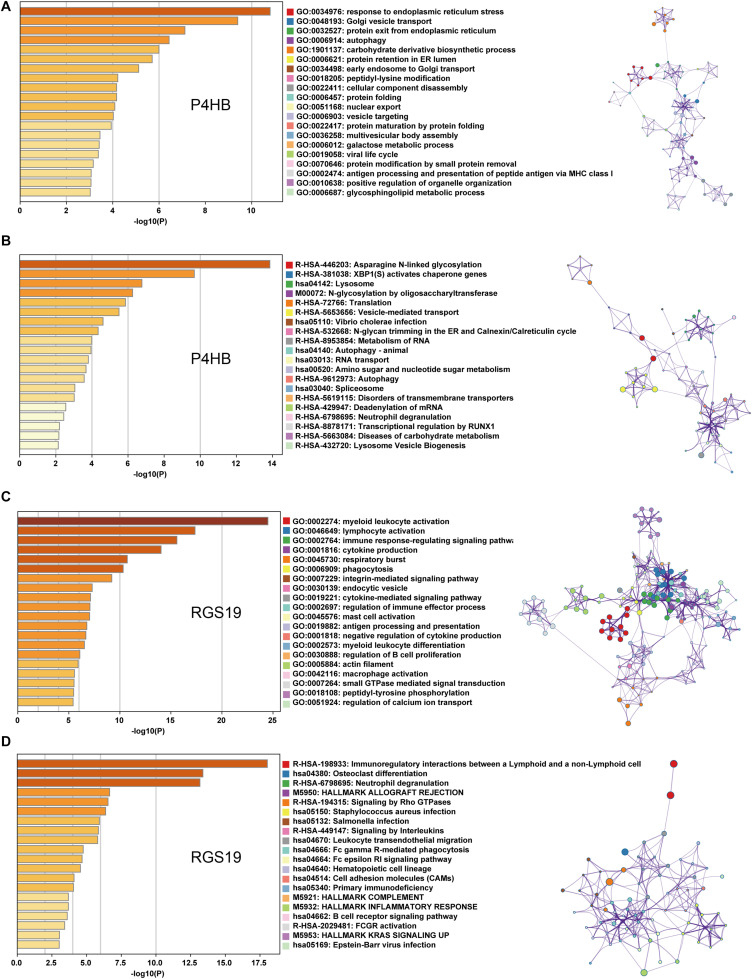
GO, KEGG and Reactome analyses of the mRNA targets of P4HB and RGS19. **(A,B)** Enrichment analyses based on GO and KEGG/Reactome pathways to predict the potential function of mRNAs targeted by P4HB. **(C,D)** Enrichment analyses based on GO and KEGG/Reactome pathways to predict the potential functions of mRNAs targeted by RGS19. The relationships among the enriched clusters from the GO and KEGG/Reactome analyses were visualized with Metascape.

**FIGURE 9:**
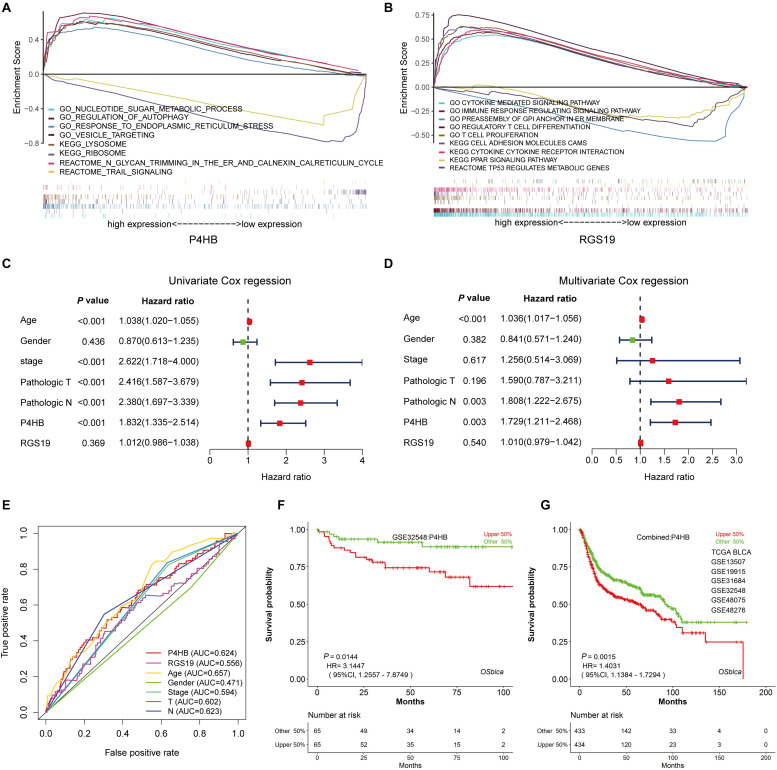
The independence of the prognostic value of P4HB from clinical characteristics. **(A,B)** GSEA using stratified P4HB and RGS19 expression levels for genes downregulated or upregulated in BUC. The GSEA results showed the correlation of the P4HB or RGS19 levels and potential biological functions in the GO, KEGG, and Reactome databases. **(C,D)** Univariate and Multivariate Cox regression analyses of the correlation between BUC patients’ OS and clinical characteristics (age, gender, neoplasm histological stage, and pathological TNM stage). **(E)** ROC curve analysis to compare the sensitivity and specificity of the prognostic values of P4HB and RGS19 and the clinical characteristics. **(F,G)** Kaplan-Meier survival curve analysis displaying the correlation of P4HB with OS for patients with BUC in the GSE32548 dataset and combined data sets (TCGA, GSE13507, GSE19915, GSE31684, GSE32548, GSE48075, and GSE48276) using the OSblca database (http://bioinfo.henu.edu.cn/).

We first confirmed the biological functions of P4HB and RGS19 using the GeneMANIA database^17^. The analysis results showed that P4HB and RGS19 had been reported to be mainly associated with lipid metabolism and GTPase activity, respectively (Figure 6). Then, we predicted the potential functions of P4HB and RGS19 in BUC using co-expression analysis methods. The Function module of LinkedOmics was used to analyze the co-expressed genes correlated with P4HB or RGS19 from 413 BUC cases in TCGA. As shown in the volcano plot (Figures 7A,B), 1619 and 3160 genes (red dots) showed significant positive correlations with P4HB (top three genes: ANAPC11, STRA13, and SLC39A7) and RGS19 (top three genes: OPRL1, ARPC1B, and OGFR), respectively, whereas 1843 and 2788 genes (green dots) showed significant negative correlations [false discovery rate (FDR) < 0.01] with P4HB (top three genes:ZSWIM6, LYRM7, and WDR36) and RGS19 (top three genes: YTHDC1, TMTC2, and NHSL1), respectively. The 50 significant gene sets positively and negatively correlated with P4HB and RGS19, as shown in the heat map (Figures 7C,D). To obtain new insights into the potential functions of P4HB and RGS19, we performed co-expression network analysis based on the Pearson correlation coefficient (| cor| ≥ 0.30, FDR < 0.01). A total of 276 and 620 protein-coding genes (PCGs) were highly correlated with P4HB and RGS19, respectively. Enrichment analyses based on GO terms, KEGG and Reactome pathways were performed to predict the potential functions of all PCGs correlated with P4HB and RGS19. The results showed that P4HB may be involved in autophagy, the response to endoplasmic reticulum (ER) stress and galactose metabolic processes. In addition, the potential functions of RGS19 may be associated with the immune response-regulating signaling pathway, cytokine-mediated signaling pathway, and inflammatory response (Figure 8). Subsequently, we performed GSEA using TCGA data to further validate the potential biological functions of P4HB and RGS19. Consistent with the results described above, stratified expression levels of P4HB and RGS19 were significantly correlated with genes associated with the nucleotide sugar metabolic process, regulation of autophagy, cytokine-mediated signaling pathway, and immune response (Figures 9A,B).

### Independence of the Prognostic Value of P4HB and RGS19 From Clinical Variables

To clarify whether P4HB and RGS19 were prognostic factors independent of other clinical variables, we performed univariable and multivariable Cox regression analyses with P4HB, RGS19, and clinical features (age, gender, cancer stage, and pathological TNM stage) as covariates. The results of the univariable and multivariate Cox regression analysis demonstrated that age, pathological N and high P4HB mRNA expression were independent and unfavorable biomarkers of OS. However, RGS19 mRNA expression had no significant relationship with OS in the univariable and multivariate Cox regression analyses (Figures 9C,D). The results of the ROC curve analysis showed that an AUC of 0.624 was achieved for P4HB, suggesting that P4HB may be an independent unfavorable prognostic biomarker of BUC (Figure 9E). The prognostic value of P4HB was further validated in the independent set (GSE32548) and combined sets (TCGA, GSE13507, GSE3164, GSE32548, and GSE48075) using the OSblca database^18^ (24). Consistent with the results of the TCGA dataset, P4HB was able to serve as a predictive factor for the prognosis of BUC (Figures 9F,G).

### Experimental Verification of P4HB in BUC Cell Lines

**FIGURE 10:**
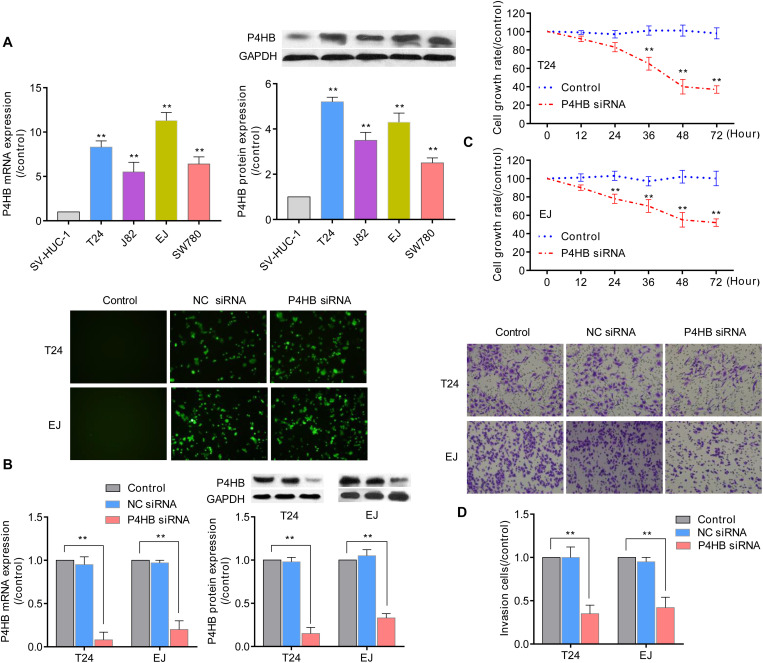
The effect of P4HB on cell invasion and viability of BUC cells. **(A)** Detection of the P4HB expression level using qRT-PCR and western blot analyses in 4 BUC cell lines (T24, J82, EJ, and SW780) and SV-HUC-1 uroepithelial cells. **(B)** After transfection with P4HB-specific siRNA for 24 h in T24 and EJ cells, the mRNA and protein expression levels of P4HB were determined by qRT-PCR and western blot analyses, respectively. Representative images of transfection are shown at 100 × magnification. **(C)** T24 and EJ cells were transfected with P4HB-specific siRNA for the indicated periods, and the cell viabilities were determined by an MTT assay. **(D)** T24 and EJ cells were transfected with P4HB-specific siRNA for 24 h and incubated for an additional 24 h in the Boyden chemotaxis chamber, and the cell invasive ability was investigated by invasion assays. Data are representative images or expressed as the mean ± SD of each group of cells from three independent experiments, **P < 0.01 vs. the SV-HUV-1 or control group.

Prolyl 4-hydroxylase, beta polypeptide expression patterns were further validated in 4 BUC cell lines (T24, J82, EJ, and SW780) and normal human uroepithelial cells (SV-HUC-1) using qRT-PCR and western blot analyses. Compared with the expression levels in SV-HUC-1 uroepithelial cells, the mRNA and protein expression levels of P4HB in the 4 BUC cell lines (T24, J82, EJ, and SW480) were significantly increased by (8.34 ± 0.75)-fold and (5.20 ± 0.23)-fold, (5.52 ± 1.10)-fold and (3.55 ± 0.35)-fold, (11.30 ± 0.94)-fold and (4.31 ± 0.44)-fold, and (6.47 ± 0.82)-fold and (2.5 ± 0.22)-fold (*P* < 0.05), respectively (Figure 10A). Since the expression level of P4HB was upregulated in bladder cancer tissues and cells, we further investigated the effect of silencing P4HB on the viability and invasion of BUC cells *in vitro*. As expected, transfection with P4HB-specific siRNA in T24 and EJ cells dramatically inhibited cell invasion and proliferation, consistent with the decreased expression levels of P4HB (Figures 10B–D), compared with the control group. The viability of T24 cells and EJ cells at 12, 24, 36, 48, and 72 h in the P4HB-specific siRNA group was (91.8 ± 3.2)% and (89.2 ± 3.0)%, (83.3 ± 5.4)% and (78.5 ± 5.0)%, (64.8 ± 7.6)% and (70.4 ± 7.2)%, (40.2 ± 8.1)% and (55.6 ± 8.3)%, and (37.5 ± 4.2)% and (52.0 ± 3.8)%, respectively (Figure 10C). The results of invasion assays demonstrated that the invasion of T24 cells and EJ cells was significantly decreased by (0.35 ± 0.12)-fold and (0.42 ± 0.14)-fold (*P* < 0.05), respectively (Figure 10D). These results further supported that regulation of P4HB may be responsible for the development of BUC.

## Discussion

Autophagy is a multistep, multistage and multifactorial complex biological process. Numerous ARGs and signaling pathways have been reported to be involved in the regulation of autophagy (25–27). Increasing evidence has shown that autophagy is an important mechanism of tumorigenesis and that interfering with autophagy signaling by targeting key ARGs may be a novel therapeutic strategy for cancer treatment. Previously, researchers have confirmed that four ARGs, including hypoxia inducible factor-1 (HIF-1α), autophagy-related 7 (ATG7), sestrin 2 (SENS2), and beclin 1 (BECN1), were associated with the cell proliferation, apoptosis and invasion of BUC cells (28–31). However, the potential clinical value of ARGs for the prognosis of patients with BUC remains unclarified.

In the present study, we first obtained 232 ARGs from the HADb and further identified 37 differentially expressed ARGs in the onset of BUC based on the TCGA database. Subsequently, a functional enrichment analysis demonstrated that these aberrantly expressed ARGs influenced apoptosis, ER stress, the cell cycle and several cancer-related pathways. We then analyzed the correlation between differentially expressed ARGs and the prognosis of patients with BUC. The results revealed that BUC patients with high expression of P4HB and RGS19 have poor OS and DFS, respectively. Thus, we next focused on investigating the biological roles of P4HB and RGS19 in BUC. The overexpression of P4HB and RGS19 was further validated in BUC using independent data sets, including those from the Oncomine and GEO databases. Moreover, cBioPortal analysis indicated that P4HB and RGS19 mRNA expression was significantly associated with mutations and alterations. Given that P4HB and RGS19 are the potential clinical values of ARGs for BUC, we further investigated the clinical significance of P4HB and RGS19 and found that these two genes were closely related to nodal metastasis and cancer stage.

Prolyl 4-hydroxylase, beta polypeptide, also known as protein disulfide-isomerase family A member 1 (PDIA1), is the main member of the PDI gene family and is identified primarily as a multifunctional protein involved in ER stress and the unfolded protein response (UPR) (32). An accumulation of UPRs in the ER leads to stress conditions and induces an autophagic response (33). Several studies have linked P4HB to various human cancers, including brain, colon, kidney and gastric cancer (34–37). Sun et al. found that P4HB could attenuate temozolomide resistance in malignant glioma via the ER stress response pathway (34). Xie et al. reported that P4HB was associated with tumor progression and the therapeutic outcome of kidney renal clear cell carcinoma (36). RGS19 is a prototypical GTPase-activating protein with multiple functions. Recent studies suggest that RGS19 can regulate autophagy by directly detecting extracellular nutrients (38, 39). Emerging studies show that RGS19 also modulates cell proliferation by forming signaling complexes with growth factor receptors. Overexpression of RGS19 could induce increased cell proliferation via enhanced Akt signaling and the deregulation of cell cycle control (40). Wang et al. reported that RGS19 suppressed Ras-induced neoplastic transformation and tumorigenesis of non-small-cell carcinoma (41). However, few reports have been published regarding the effects and mechanisms of action of P4HB and RGS19 in BUC. Therefore, we further investigated the potential functions of P4HB and RGS19 in BUC using co-expression network analysis and GSEA. We found that P4HB and RGS19 may influence the progression and prognosis of BUC by regulating the ER stress response, cytokine-mediated signaling pathway and inflammatory response. The development and progression of bladder cancer involves multiple factors, such as clinicopathological features. We further analyzed whether P4HB and RGS19 are independent poor prognosis factors in BUC using multivariate Cox proportional hazards regression analysis. The results revealed that P4HB but not RGS19 is an independent unfavorable biomarker from conventional clinical factors (age, gender, cancer stage, and pathological TNM stage), indicating that P4HB may potentially enable clinicians to discriminate high-risk patients from low-risk patients with identical clinical characteristics for rationalizing treatment decisions. We investigated the biological effect of P4HB in BUC cells *in vitro.* We found that the mRNA and protein expression of P4HB in BUC cells was upregulated compared with the expression in SV-HUC-1 uroepithelial cells, and knockdown of P4HB dramatically inhibited the cell invasion and proliferation of BUC cells. These preliminary *in vitro* experimental results confirmed that P4HB may be associated with the development and progression of bladder cancer. Previous studies have demonstrated that PH4B can induce malignant tumor cell proliferation, invasion and metastasis by regulating hypoxia inducible factor-1α (HIF-1α) expression and the MAPK signaling pathway (42, 43). In our present study, we found that P4HB expression was positively associated with the expression levels of ANAPC11, STRA13, and SLC397A and negatively associated with the expression levels of ZSWIM6, LYRM7, and WDR36 (Figure 7A). However, few reports have demonstrated that these genes, which are closely related to PH4B, are associated with the development of BUC. Interestingly, we performed GSEA to predict the potential biological functions of P4HB expression-related genes and found that these genes were involved in the nucleotide sugar metabolic process, regulation of autophagy, the response to ER stress and vesicle targeting (Figure 9A), suggesting that P4HB may provide some new insights into the molecular mechanism of BUC and novel treatment targets.

## Conclusion

In this study, we identified P4HB from hundreds of candidate ARGs in large-scale BUC samples; P4HB can be used as a diagnostic and prognostic marker for patients with BUC and can also provide a better understanding of the regulatory mechanisms of autophagy involved in the development of BUC. Moreover, there are still some limitations of the present study that should be acknowledged. We will perform further experimental research *in vitro* and *in vivo* to investigate the precise functions and mechanisms of P4HB in the regulation of autophagy-mediated tumorigenesis in BUC.

## Data Availability Statement

The datasets presented in this study can be found in online repositories. The names of the repository/repositories and accession number(s) can be found in the article/supplementary material.

## Author Contributions

LL and CZ designed the experiments. LL, WX, and FZ performed the experiments. LL and WX drafted the manuscript. LL, TH, YF, and JY conducted the data analysis and interpreted the results. LL and CZ revised the manuscript. All authors reviewed and approved the manuscript.

## Conflict of Interest

The authors declare that the research was conducted in the absence of any commercial or financial relationships that could be construed as a potential conflict of interest.

## Footnotes

1 http://www.autophagy.lu

2 https://portal.gdc.cancer.gov

3 http://www.geneontology.org

4 http://www.kegg.jp

5 https://reactome.org

6 http://www.metascape.org/

7 http://www.broad.mit.edu/gsea

8 http://gepia.cancer-pku.cn

9 www.oncomine.org

10 www.ncbi.nlm.nih.gov/gds

11 http://ualcan.path.uab.edu/analysis.html

12 http://cbioportal.org

13 http://www.linkedomics.org/login.php

14 http://genemania.org

15 http://sidirect2.rnai.jp

16 https://www.proteinatlas.org

17 http://genemania.org

18 http://bioinfo.henu.edu.cn/
